# Refractive index engineering through swift heavy ion irradiation of LiNbO_3_ crystal towards improved light guidance

**DOI:** 10.1038/s41598-017-11358-y

**Published:** 2017-09-07

**Authors:** Chen Chen, Lilong Pang, Qingming Lu, Lei Wang, Yang Tan, Zhiguang Wang, Feng Chen

**Affiliations:** 1 School of Physics, State Key Laboratory of Crystal Materials, Key Laboratory of Particle Physics and Particle Irradiation (Ministry of Education), Shandong University, 250100 Jinan, China; 2 Institute of Modern Physics, Chinese Academy of Sciences, 730000 Lanzhou, China; 3 School of Chemistry and Chemical Engineering, Shandong University, 250100 Jinan, China

## Abstract

Swift heavy ion irradiation has been widely used to modify refractive indices of optical materials for waveguide fabrication. In this work, we propose refractive index engineering by swift heavy ion (Ar) irradiation via electronic energy deposition to construct waveguides of diverse geometries in LiNbO_3_ crystal. The feasibility to modulate the refractive index of LiNbO_3_ crystal at variable depths through electronic energy depositions of argon ions at different energies has been experimentally explored. The surface and cladding-like optical waveguides with thicknesses of ~13, ~36 and ~23 μm have been produced by using swift Ar ion irradiation at single energy of ~120, ~240, and double energy of (120 + 240) MeV, respectively. The fabricated waveguides are capable of effective waveguiding in single and multiple modes at 1064 nm, which enables efficient guided-wave second harmonic generation at room temperature. This work paves the way to produce waveguides with diverse geometries in dielectric crystals through electronic damage of multiple swift heavy ion irradiation.

## Introduction

Energetic ion beam processing techniques, such as ion implantation, swift heavy ion irradiation, focused ion beam irradiation, have been intensively applied to modify the physical, chemical, electrical and optical properties of numerous materials^1–8^. It has been demonstrated that the incident ions could induce a refractive index change of the target crystal to produce optical waveguide structure^7, 8^. Through the implantation or irradiation of ions with diverse species, fluences, and energies, waveguide structures have been fabricated in a large number of optical materials with desirable geometries and refractive index distributions^7^. Swift heavy ion irradiation and traditional ion implantation have essential distinction in the mechanism of waveguide formation^8^. For light ion implantation, the refractive index modification is induced chiefly through the structural damage caused by elastic nuclear collisions between incident ions and target atoms (correlated to nuclear stopping power *S*_n_), which happens mainly at the end of ion’s range. In the process of swift heavy ion irradiation, which is commonly implemented with ions of larger masses (typically with atomic number no less than 8, e.g. O, F, Ar, Kr) and at the energy over 1 MeV/amu, the damage attributed to electronic energy deposition (related to electronic stopping power *S*_e_) plays the crucial role in the modification of refractive indices. The relatively large value of *S*_e_ enables the reduction of ion fluence to the magnitude of 10^11^ to 10^13^ cm^−2^, which is significantly lower than the fluence required in light ion implantation (typically in the order of 10^16^ to 10^17^ cm^−2^)^9^. Except for higher efficiency in waveguide construction, irradiation with lower fluence has additional advantages of significantly reduced processing time and also preserving the original properties (e.g. optical nonlinearity, lasing, electro-optical property) of target materials^10^. Moreover, the thicker optical barrier formed by ion irradiation could effectively avoid light tunneling into substrate and thus offer better confinement^11^. Nevertheless, to precisely modulate the refractive index via electronic energy deposition is challenging to realize since the dependence of *S*_e_ on the incident energy is not as remarkable as that of *S*_n_.

Lithium niobate (LiNbO_3_) is famous for its large nonlinear coefficient as well as excellent acoustic-optical and electro-optical properties^12–15^. Numerous applications based on LiNbO_3_ have been realized including electro-optic modulation, optical switching, frequency doubling and optical parametric oscillation/amplification^11–20^. LiNbO_3_ is also an ideal choice as a platform for waveguide fabrication^21^. Guiding structures with diverse geometries have been produced in LiNbO_3_ through a wide variety of techniques, such as ion exchange, Ti diffusion, ion implantation/irradiation, and ultrafast laser inscription^22–27^.

In previous works, refractive index of LN has been modulated by accumulation of nuclear-collision induced damage in multiple ion implantations to obtain a desirable step-like profile^28, 29^. The modifications of LN refractive indices through electronic energy deposition have been investigated in single swift heavy ion (e.g. Cl, Ar, Kr, Xe) irradiation at ultralow fluence (10^11^ to 10^12^ cm^−2^)^11, 30, 31^. In this work, we propose refractive index engineering by double swift heavy ion (Ar) irradiation to construct a buried cladding-like index distribution in LiNbO_3_ crystal. The feasibility to modulate the refractive index of LiNbO_3_ crystal at variable depths through electronic energy depositions of argon ions at different energies has been experimentally explored. The fabricated waveguides with diverse geometry are capable of effective waveguiding in single and multiple modes at 1064 nm, which enables efficient guided-wave second harmonic generation at room temperature. Based on this research, photonic devices with various refractive index distributions are expected to emerge through the superposition of electronic damage in multiple swift heavy ion irradiations.

## Results

### Argon ion irradiation onto LiNbO_3_

**Figure 1:**
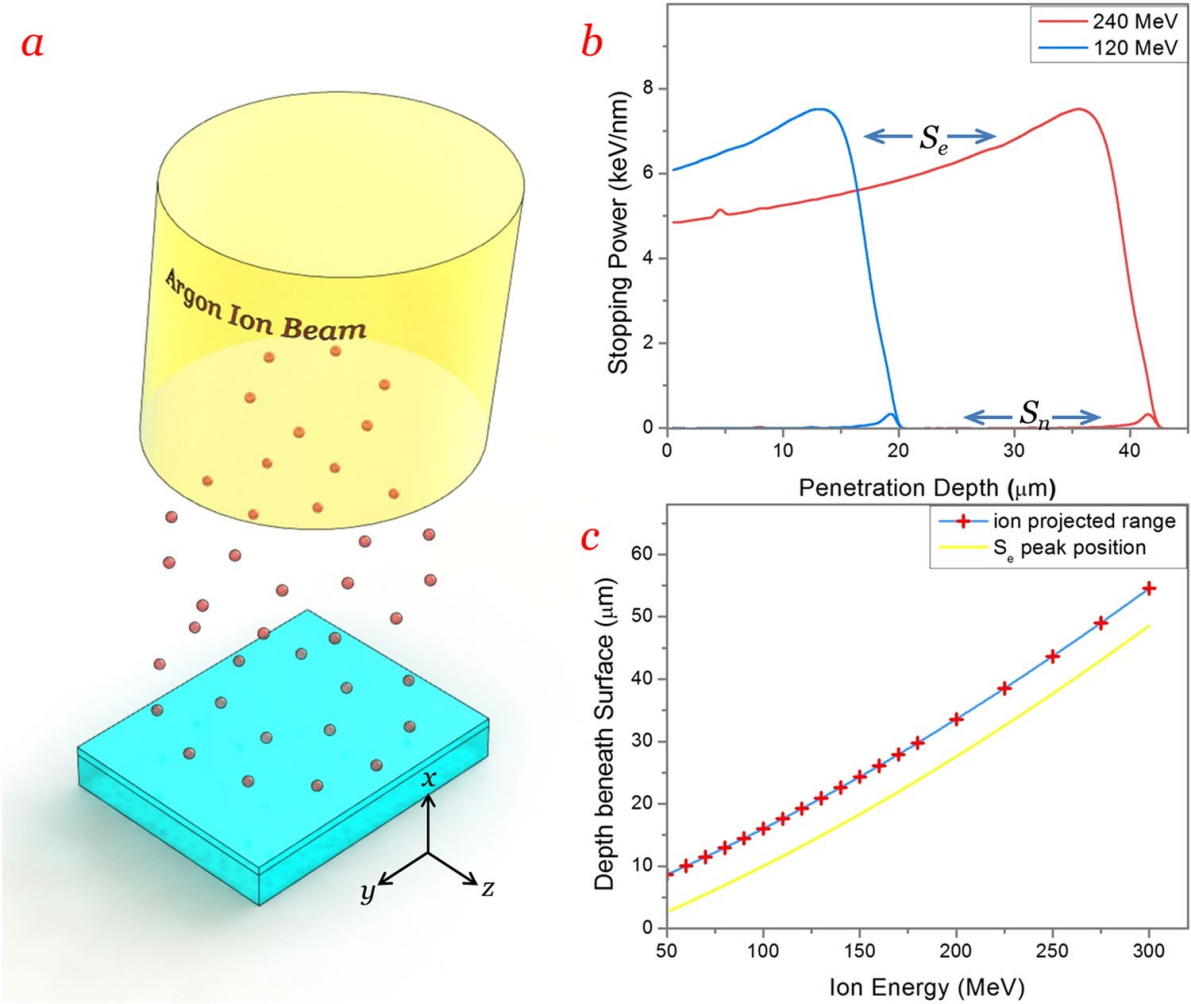
Argon ion irradiation onto LiNbO_3_. (**a**) Schematic plot of argon ion irradiation onto x-cut LiNbO_3_. (**b**) Computed electronic (*S*_e_) and nuclear (*S*_n_) stopping powers of argon ions versus penetration depth at energy of 120 and 240 MeV respectively. (**c**) Projected range and peak position of *S*_e_ for argon ions into LiNbO_3_ as functions of incident energy.

The irradiation of argon ions onto *x*-cut LiNbO_3_ samples is illustrated in Fig. 1a. Interactions between incident argon (Ar^12+^) ions with energies ranging from 50 to 300 MeV and the target LiNbO_3_ crystal were predicted using the code of Stopping and Range of Ions in Matter 2013 (SRIM 2013)^32^, which is based on Monte Carlo approach. The energy of incident argon ions was lost mainly by two mechanisms: electronic excitations (related to *S*_e_) and nuclear collisions (related to *S*_n_). Figure 1b exhibits computed *S*_e_ and *S*_n_ stopping powers of argon ions at energies of 120 and 240 MeV as functions of penetration depth. We find that the peak value of *S*_e_ is a constant (~7.5 keV/nm) irrelevant with the incident energy. The peak position of *S*_e_ (corresponding depth) increases with the incident energy. For example, the *S*_e_ reaches its peak value at the penetration depth of ~13 μm for the energy of 120 MeV, at ~36 μm for 240 MeV. Compared with *S*_e_, the value *S*_n_ is negligible, which has only a little jump at the end of ion projected range. The distance between the peak positions of *S*_n_ and *S*_e_ is also irrelevant with the incident energy, which is always around 6 μm.

Figure 1c shows the projected range (approximately equals peak position of *S*_n_ curve) of Ar ions into LiNbO_3_ as a function of the incident energy (blue curve with red cross symbols), the curve for the peak position of *S*_e_ versus energy is also drawn (in yellow color), which is below the former curve by ~6 μm. Since the electronic damage (correlated to *S*_e_) is dominant throughout the whole ion range, it can be utilized as the main solution to modify the refractive index of LiNbO_3_ crystal. As reported by Olivarez *et al*., the refractive index change (decrease) of LiNbO_3_ is related to the value of *S*_e_ when the amorphous threshold (~5.5 keV/nm for LiNbO_3_) is exceeded^27^. According to the curves, we can create an optical barrier with index decrease by electronic damage (instead of nuclear one) at variable depth (in accordance with the peak position of *S*_e_) by changing the incident energy of argon ions to produce a waveguide layer. Besides, two or more optical barriers at different depth can also be built by multiple irradiations with different energies, in which the buried cladding-like waveguide structure would be formed between the barriers.

### Guiding properties of irradiated layers

**Figure 2:**
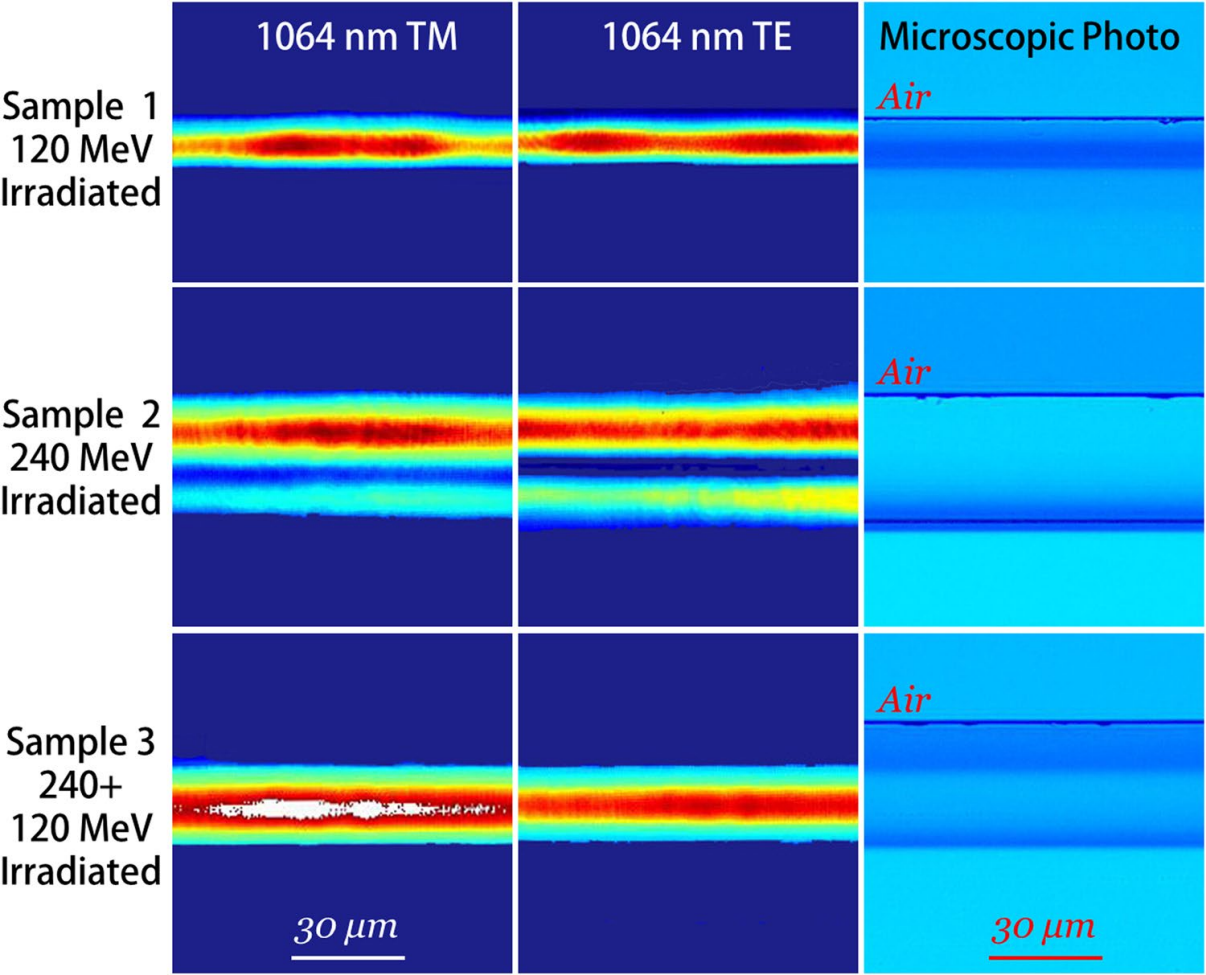
Guiding properties of irradiated layers. Measured intensity distributions of guided TM modes (left column) and TE modes (center column) at the wavelength of 1064 nm for layers irradiated under different conditions. (right column), Cross-sectional microscopic photographs for different layers.

In this work, two LiNbO_3_ samples were irradiated at the energy of 120 and 240 MeV respectively, with another sample irradiated at double-energy of 240 and 120 MeV successively. The microscopic photographs were taken from the polished end faces at transmission mode, as depicted in Fig. 2 right column. As one can see, the surface layer structures with different thickness were formed after single ion irradiation (Fig. 2 right column, top and middle). A cladding-like layer can be seen apparently in sample 3 (Fig. 2 right column, bottom). The total thickness measured under microscope is ~19, ~42, ~42 μm respectively for these three samples, which shows well consistency with the calculated ion projected range (Fig. 1c). The position of optical barrier, is measured to be ~13 and ~36 μm, consistent with the peak position of *S*_e_.

The guiding properties of irradiated layers were investigated using a typical end face coupling arrangement at the wavelength of 1064 nm. Figure 2 (left and center columns) displays the intensity distributions of guided TM (ordinary index, *n*_o_) and TE (extraordinary index, *n*_e_) modes. It can be found that the light fields are well confined in waveguide layers. The guided modes were supported in higher order (TM_1_, TE_1_) for the waveguide irradiated by 240 MeV due to its large thickness. For other waveguides, the guided wave propagated in fundamental modes (TM_0_, TE_0_). The light energy seems to be more concentrate in the cladding-like structure, especially along TM polarization.

The propagation losses were determined by direct measurement of the input and output light powers through the waveguides, with Fresnel reflections at air/waveguide interfaces and coupling losses of light beam profiles and waveguide modes taken into account. Here, the coupling losses *α*_*c*_ (4.09, 4.41 and 3.74 dB for samples 1–3) were roughly estimated by the equation below^33^:

1 $$\eta =\frac{2ax}{{a}^{2}+{x}^{2}}\cdot \frac{2ay}{{a}^{2}+{y}^{2}}$$

2 $${\alpha }_{c}=-10\,{\rm{l}}{\rm{o}}{\rm{g}}\,\eta $$

where *a* represents the diameter of the focused light spot (~20 μm), *x* and *y* are the height (~13, ~36 and ~23 μm for sample 1–3) and width (~90 μm) of guided mode. The propagation loss for sample 1, 2 and 3 is determined to be 3.98, 3.66, 3.01 dB/cm, respectively, under TE polarization at 1064 nm. Compared with the value of cladding waveguide, the propagation losses of surface waveguide (samples 1 and 2) are considerably higher, which may result from the additional scattering loss induced by surface roughness of the samples.

### Reconstructed refractive index profiles

**Figure 3:**
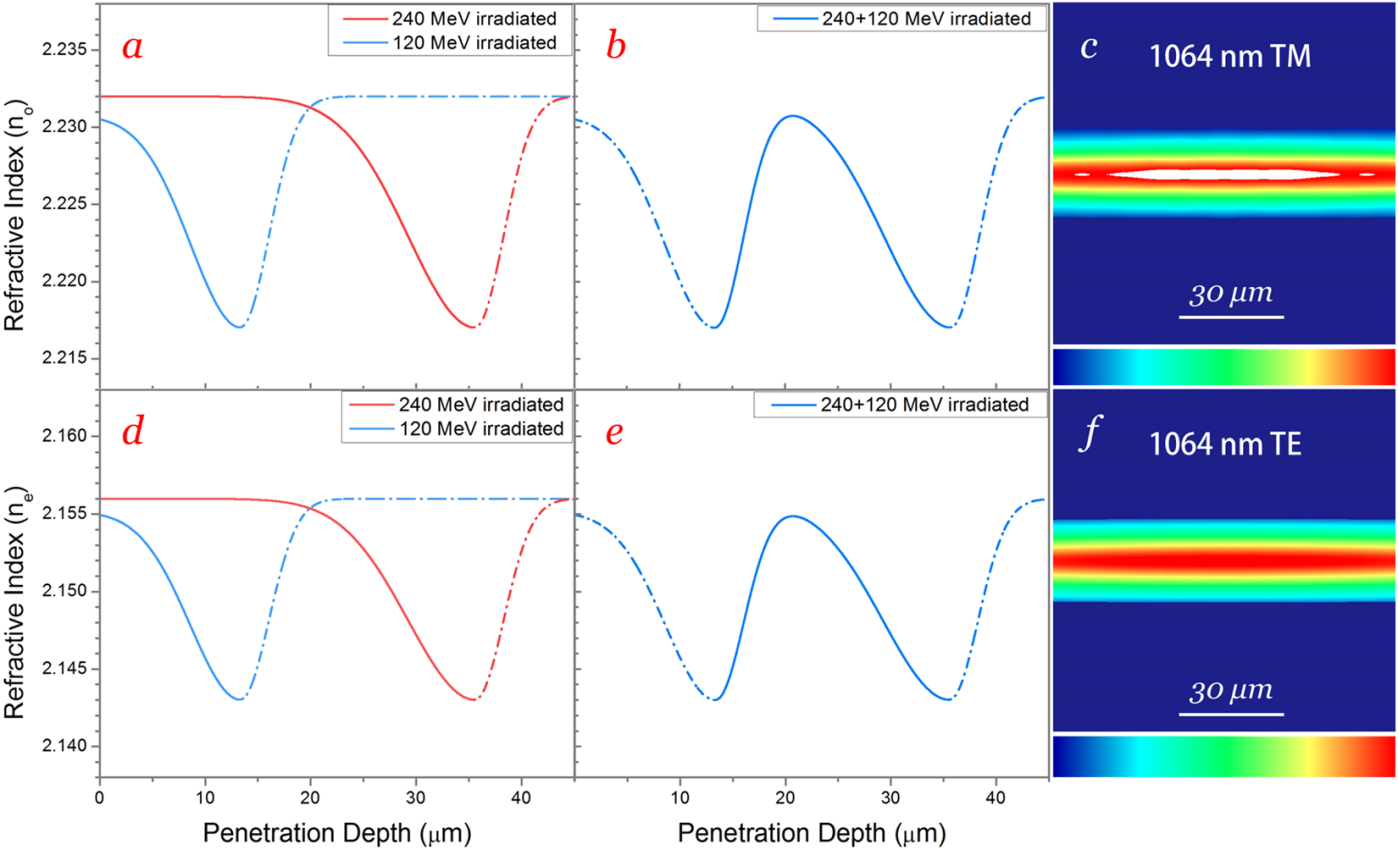
Reconstructed refractive index profiles. Ordinary and extraordinary refractive index profiles for single (**a**,**d**) and multiple (**b**,**e**) energy irradiated LiNbO_3_ crystals. Simulated TM (**c**) and TE (**f**) guided modal profiles of cladding-like layer at 1064 nm.

The ordinary refractive index profiles at 1064 nm for single energy irradiated LiNbO_3_ crystals were reconstructed, as illustrated in Fig. 3a, the solid parts of curves represent the effective waveguiding regions. The basic shape of the profile is similar to that of a 200 MeV argon ion irradiated SLN reported by Huang *et al*., which was determined by dark modes and smoothed via inversed WKB method^31, 34^. In current profiles, depths of optical barrier were determined in accordance with peak positions of *S*_e_, while the refractive index contrasts *Δn* (~0.015) between surface and barrier were given by the following equation^35^:

3 $${\rm{\Delta }}n=\frac{{\sin }^{2}{{\rm{\Theta }}}_{m}}{2n}$$

where *n* (2.232) is refractive index of substrate, and *Θ*_m_ the maximum incident angle (15°), which is the largest angle allowed between incident light beam and end-face normal of sample. It should be noted that the existing form of argon (compound or element) would not influence the stability of waveguide because the argon atoms are distributed at the end of projected range, which is out of waveguide region. Since the peak value of *S*_e_ for the cases of 120 and 240 MeV is identical, it is reasonable to assume that the index of barrier is at the same level for such samples. However, *Θ*_m_ of 120 MeV irradiated sample is smaller (14.2°), indicating a possible index decrease (~0.0015) in the surface with respect to the virgin crystal. The phenomenon can be explained by the surface value of *S*_e_, for 120-MeV irradiated sample, the value (~6 keV/nm) is above the amorphous threshold (~5.5 keV/nm) at surface, where single impact of incident ions will create amorphous tracks. Whilst for 240 MeV-irradiated sample, the value (~5 keV/nm) is below the threshold for single ion impact, the surface index modification is mainly due to the synergy effect of a number of ions (damage overlap)^36^. The ordinary refractive index for the cladding-like waveguide constructed by multiple irradiations, as shown in Fig. 3b, was obtained from the superposition of index decreases for single energy irradiated ones, given by the equation below:

4 $$n(x)={n}_{sub}-{\rm{\Delta }}n(x)$$

5 $${\rm{\Delta }}n(x)={\rm{\Delta }}{n}_{1}(x)+{\rm{\Delta }}n{}_{2}(x)$$

where Δ*n*_1_(*x*) and Δ*n*_2_(*x*) represent the index decrease at the depth *x* in sample 1 and 2 respectively.

Based on this index profile, we calculated the modal intensity distribution using the software Rsoft Beam PROP 8.0^©^, which is operated upon the finite difference beam propagation method (FD-BPM)^37^. As presented in Fig. 3c, the simulated TM mode for the cladding-like waveguide at 1064 nm has well agreement with the measured one (Fig. 2 left column, bottom), demonstrating the reasonability of such reconstructed index profile. The extraordinary refractive indices were reconstructed by same method, as exhibited in Fig. 3d and e. Similarly, good agreement between the simulated TE mode (Fig. 3f) and the measured one (Fig. 2 center column, bottom) has demonstrated its reasonability.

### Guided-wave second harmonic generation

**Figure 4:**
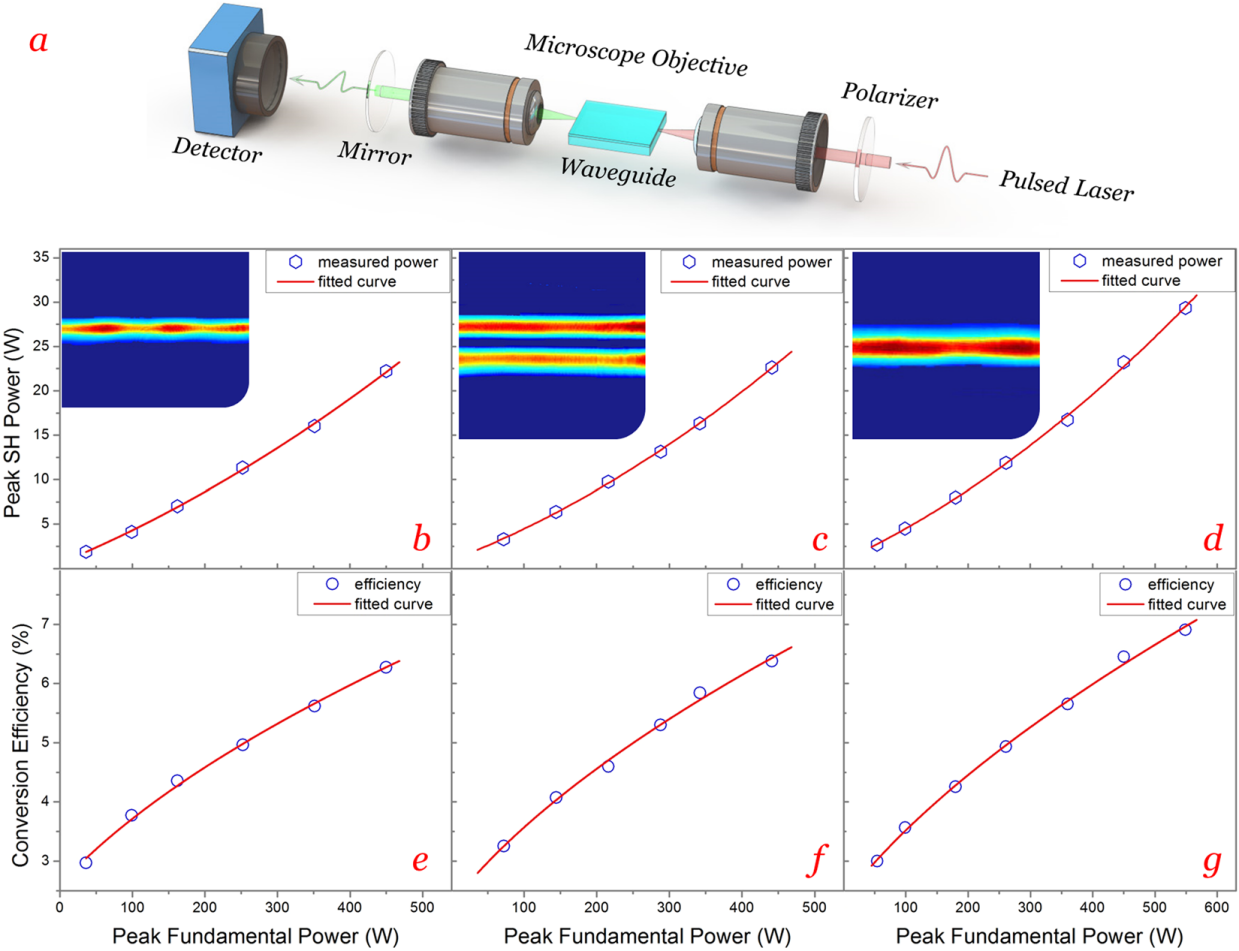
Guided-wave second harmonic generation. (**a**) Experimental setup for guided-wave SHG in pulsed regime. Second harmonic powers (**b**,**c**,**d**) and conversion efficiencies (**e**,**f**,**g**) as functions of the launched fundamental powers measured from sample 1 to 3. (insets) measured SH modal profiles from sample 1 to 3.

The experiments of guided-wave second harmonic generation (SHG) were carried out on the basis of end-face coupling method, as sketched in Fig. 4a. The fundamental wave was a pulsed laser at 1064 nm polarized along TM direction; while the SH wave was generated at 532 nm along TE polarization under non-critical phase matching (TM^ω^ → TE^2ω^) at room temperature (293 K)^38^. Figure 4b–d present the second harmonic powers as functions of the launched fundamental powers measured from sample 1 to 3. One can see the harmonic powers increase nonlinearly with fundamental powers, which are close to quadratic curves as fitted. The maximum peak powers of guided harmonic pulses are measured to be 22.4, 22.8, and 29.5 W for sample 1, 2, and 3. As given in Fig. 4e–g, the conversion efficiencies increase with fundamental powers as well, with the maximum efficiencies determined to be 6.28%, 6.42%, and 6.93% for sample 1, 2, and 3 respectively.

The cladding-like waveguide (sample 3) possesses the best performance of SHG, which may result from its advantageous construction and index distribution. On one hand, the buried guiding layer effectively prevents the scattering of light by surface roughness; on the other hand, the superposition of refractive index enables a more concentrate distribution of light energy. The SH modal profiles are shown in the insets of Fig. 4. As we can see, the generated harmonic wave propagated in higher order mode (TE_1_) inside the 36 μm-thick waveguide, which would certainly degrade its SHG performance. Nevertheless, the propagation of multiple modes at 532 and 1064 nm may indicate the potential application of the 240 MeV-irradiated waveguide at larger wavelength (e.g. mid-infrared band).

In general, the SHG performances within such waveguides are fairly well via non-critical phase matching at room temperature, showing that the nonlinear optical properties of LiNbO_3_ crystal have been preserved substantially after the irradiation of argon ions. By providing a suitable temperature control of the nonlinear optical process, the performances of SHG could be further improved and comparable with other nonlinear optical waveguide systems.

## Discussion

We have explored the feasibility of modulating the refractive indices of x-cut LiNbO_3_ crystal in variable depths by electronic energy depositions of irradiated argon ions. As demonstrations, surface and cladding-like waveguides with thicknesses of ~13, ~36, ~23 μm have been constructed in practice using the single and double irradiations at energy of 120 and 240 MeV. The fabricated constructions are capable of effective wave guiding in single and multiple modes at 1064 nm. According to the variations of electronic stopping power, we have reconstructed ordinary and extraordinary refractive index profiles in a reasonable manner. Second harmonic generation has been realized within such waveguide structures via non-critical phase matching at room temperature.

In current experiments, the cladding-like waveguide is found to be superior to surface ones in guiding properties and SHG performance, which is likely due to its better confining refractive index distribution and buried structure without surface scattering. On the other side, the surface waveguide has advantages in generating and receiving evanescent field, which enables the potential applications in chemical or biological photonic sensing. The modulation of refractive index in variable depth by swift heavy ion irradiation with tailored energies could be flexibly implemented by other ion species and on various materials, to realize waveguide construction with diverse geometries and functions. And the superposition of electronic damage in multiple ion irradiations supplies the possibility to construct new-type refractive index distribution of the structures. Although it is still challenging for swift heavy ion irradiation to independently and directly build two dimensional and more complicated configurations for photonic applications, by combining such methods with surface patterning techniques, such as lithography, chemical etching, diamond dicing, laser ablating, etc., one could expect the achievement of versatile integrated photonic devices in novel constructions.

## Methods

### Fabrication of waveguides

Three pieces of *x*-cut LiNbO_3_ samples, with dimension of 10(*y*) × 8(*z*) × 1(*x*) cm^3^, were optically polished at top surface. The irradiations of argon ions were performed at Heavy Ion Research Facility in Lanzhou (HIRFL). The initial incident ion energy was 247 MeV, which was reduced by a 3 μm-thick aluminum foil to 240 MeV, or reduced by a 34.2 μm-thick aluminum foil to 120 MeV. Two samples (sample 1 and 2) were irradiated at the energy of 120 and 240 MeV respectively, with another sample (sample 3) irradiated at 240 and 120 MeV successively. For each irradiation, the fluence was set to be 1 × 10^12^ ions cm^−2^. During the irradiation, current density of ion beam was kept at a level (<60 nA/cm^2^).

### Investigation of guiding properties

Guiding properties were investigated using a typical end-face coupling arrangement. The input light at 1064 nm was polarized by a half-wave plate and coupled into waveguides by a microscope objective lens (25 × N.A. = 0.4). The output radiation was collected by another lens and recorded by a detector (CCD camera) at the end of light path.

### Guided-wave second harmonic generation

The experiments of SHG were carried out with the same end-face coupling arrangement. A Q-switched solid-state Nd:YAG laser operating at 1064 nm with pulse duration of ~11.05 ns, repetition rate of ~5 kHz, and pulse energy of ~80 μJ acted as fundamental light source. The fundamental power was controlled by an attenuator. A mirror with high reflectivity (HR) at 1064 nm and high transmittance (HT) at 532 nm was positioned behind the out-coupling lens to filter the residual fundamental wave out.
